# Posture Used in fMRI-PET Elicits Reduced Cortical Activity and Altered Hemispheric Asymmetry with Respect to Sitting Position: An EEG Resting State Study

**DOI:** 10.3389/fnhum.2017.00621

**Published:** 2017-12-18

**Authors:** Chiara Spironelli, Alessandro Angrilli

**Affiliations:** ^1^Department of General Psychology, University of Padova, Padova, Italy; ^2^Institute of Neuroscience, National Research Council (IN-CNR), Rome, Italy

**Keywords:** fMRI, supine position, body posture, hemispheric asymmetry, resting state, EEG

## Abstract

Horizontal body position is a posture typically adopted for sleeping or during brain imaging recording in both neuroscience experiments and diagnostic situations. Recent literature showed how this position and similar ones with head down are associated to reduced plasticity, impaired pain and emotional responses. The present study aimed at further understanding the decrease of cortical activity associated with horizontal body position by measuring high-frequency EEG bands – typically associated with high-level cognitive activation – in a resting state experimental condition. To this end, two groups of 16 female students were randomly assigned to either sitting control (SC) or 2-h horizontal Bed Rest condition (hBR) while EEG was recorded from 38 scalp recording sites. The hBR group underwent several body transitions, from sitting to supine, and from supine to sitting. Results revealed a clear effect of horizontal posture: the hBR group showed, compared to its baseline and to SC, reduced High-Beta and Gamma EEG band amplitudes throughout the 2-h of hBR condition. In addition, before and after the supine condition, hBR group as well as SC exhibited a greater left vs. right frontal activation in both EEG bands while, on the contrary, the supine position induced a bilateral and reduced activation in hBR participants. The cortical sources significantly more active in SC compared with hBR participants included the left Inferior Frontal Gyrus and left Insula. Results are discussed in relation to the differences among neuroimaging methods (e.g., fMRI, EEG, NIRS), which can be partially explained by posture-induced neural network changes.

## Introduction

The milestone technique of the neuroscience era is represented by neuroimaging methods, in particular the functional magnetic resonance imaging, which allowed researchers to capture the activity of the human brain *in vivo*. In a few decades, neuroimaging research provided a quite impressive number of studies that clarified the anatomo-functional correlates underlying the main cognitive human domains (e.g., Christoff et al., 2004; Frackowiak, 2004; Hill and Linden, 2013). Although the key role of functional neuroimaging research is undeniable, very recent and specialized literature started to address an important and critical issue associated with one of the main characteristics of neuroimaging techniques. Indeed, almost universally, the experimental session of a typical fMRI acquisition requires participants to lie supine (Thibault et al., 2014). This simple, and apparently irrelevant technical prerequisite postulates that the execution of whichever task or cognitive processing is not affected by our body posture. Indeed, we spend most of our awake life in standing or sitting position, and certainly in most of cognitive experiments carried out in psychological labs, using behavioral measures or EEG, TMS, and so on, participants always sit upright, but in fMRI studies all participants are tested and their brain activity is measured while their bodies lay down in a supine/horizontal position.

Some critical cues on this issue arise from a very different framework, i.e., past EEG literature on microgravity simulated through Head Down Bed Rest with -6 degrees inclination. Bed Rest studies suggested that during supine posture many physiological processes are significantly altered (Fritsch-Yelle et al., 1994), including cortical activity (Vaitl and Gruppe, 1992; Vaitl et al., 1996; Spironelli et al., 2016; Spironelli and Angrilli, 2017), pain processing (Spironelli and Angrilli, 2011; Fardo et al., 2013), emotional responses (Messerotti Benvenuti et al., 2013), and brain plasticity (Messerotti Benvenuti et al., 2011). According to Vaitl et al. (1996), in an open-eyes, resting-state task, horizontal as well as head down bed rest compared with standing and seated position elicited an increase of spontaneous low-frequency EEG rhythms, i.e., the delta and theta bands. Cheron et al. (2006) carried out an experiment over the course of three space flights, to quantify the power of the 10-Hz activity in two distinct physiological states: eyes open and eyes closed. The authors found that, in the absence of gravity, the power of the spontaneous 10-Hz oscillation recorded in the eyes-closed state increased in the parieto-occipital (alpha rhythm) and sensorimotor areas (mu rhythm). Instead, during a low-level visuo-attentional task an alpha ERD potentiation was consistently observed in microgravity, due to the activation of the bilateral motor cortex together with the activation of both the cerebellum and the vestibular system (Cebolla et al., 2016). The authors interpreted the activation of this cortical/subcortical network as necessary to the readjustment and maintenance of an appropriate body posture as well as the correction and error signals for postural stabilization while free-floating.

Concerning the functional meaning of these low-frequency bands (EEG alpha, delta, and theta), depending on the setting, paradigm, task and stimuli, an increase of amplitude in these bands may index either cortical activation or inhibition. During engagement of the participant in perceptual tasks, an increase of slow band amplitude was associated with better performance (Henry et al., 2014; Arnal et al., 2015; Yan et al., 2017). The same EEG bands have been studied and modeled as biomarkers of optimal performance in sport (for a review, see Cheron et al., 2016). Other studies with different paradigms suggested that delta and theta bands mark cortical inhibition, and are therefore considered markers of brain sufferance or pathological condition when they appear in the waking brain (Gloor et al., 1977; De Jongh et al., 2003; Spironelli et al., 2006, 2008, 2011; Penolazzi et al., 2008; Spironelli and Angrilli, 2009).

On the basis of this evidence, Vaitl et al. (1996) suggested that the body position may also induce a cognitive impairment. Very few recent studies addressed this topic in order to identify how posture affects the human brain activity. Chang et al. (2011) investigated the association between autonomic response (using the HRV index) and cortical activity (measured with EEG) by manipulating participants’ body position (supine vs. upright). Changing position from upright to supine, authors found both a significant increase of slow frequency (i.e., delta and theta) rhythms – in agreement with past studies on simulated microgravity (Vaitl and Gruppe, 1992; Vaitl et al., 1996) – and a significant decrease of high-frequency (i.e., beta and gamma) bands. That study, however, has been carried out while participants kept their eyes closed, a condition which increases low frequency activity (especially EEG alpha band) and is not comparable with ecological human condition of sitting/upright position typically held with open eyes. Messerotti Benvenuti et al. (2013) first suggested that research carried out with fMRI and supine subjects could provide results differing substantially from those achieved in sitting participants. A following study (Thibault et al., 2014) stressed the importance of considering posture an important condition, when neuroimaging techniques are used to investigate the mechanisms underlying the human brain functioning. In that research, authors compared four different postures (i.e., supine, 45° inclined, sitting and standing) during resting state and mental counting task (both open- and closed-eyes). Unlike Chang et al. (2011), a high-density EEG system has been used, but, in agreement with that study, significant decrease of high-frequency bands was found in supine rather than upright posture, regardless of task (none or mental counting) and eyes (open or closed). In the Thibault et al.’s (2014) study, a within-subjects repeated measure design has been used: to this end, only 30 s of EEG activity for each condition has been recorded, and participants maintained each position for very limited time (i.e., 150 s). Currently, there is no clue on the possible difference between transient and static body transitions on the observed cortical modifications. Depending on the underlying physiological mechanism, the cortical inhibition may result from rapid body position change (e.g., mediated pre-eminently by arterial baroreceptors sensing blood pressure shifts) or, alternatively, by a physiological (e.g., hormonal) condition slowly building up across time.

**FIGURE 1:**
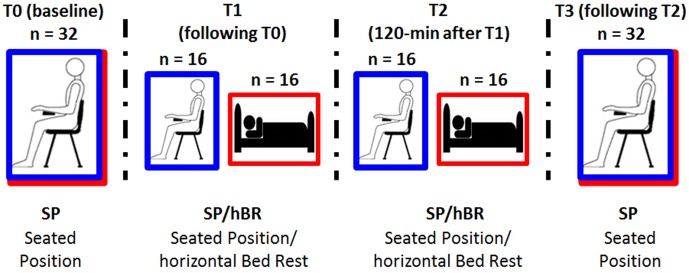
Schematic representation of the experimental design. Participants were randomly assigned to experimental [i.e., horizontal Bed Rest (hBR); red line] or control condition [i.e., Sitting Control (SC); blue line], and underwent four EEG recordings along the 2.5-h EEG session.

The present study add to our current knowledge on the field, by analyzing the effects of body posture in a between-subjects experimental paradigm, in which two samples of healthy young women underwent repeated measures either in seated control (SC group) or supine position [horizontal Bed Rest (hBR group)]. We avoided the pure within-subjects design adopted in other experiments, in order to avoid potential long-term carry over effects of the supine position over a control, post-supine condition. High-frequency cortical activity (i.e., High-Beta and Gamma bands) was analyzed, and four EEG measures were collected while participants were open-eyed relaxed: first, both group started in the same seated interval (T0), then the experimental group laid down while the control group remained sitting (T1), in order to investigate mid-term effects; measures were repeated in the same position after 120 min (T2) and finally, both groups have been tested again in the sitting interval (T3) (see **Figure 1** for procedure details). The first measure allowed us to compare the EEG activity of different groups at baseline to exclude *a priori* EEG differences in the random assignment to SP or hBR group, the second measure (T1) permitted to highlight the effects of seated vs. supine/horizontal position on the resting state EEG activity soon after the body transition. In addition, T2 and T3 measures were aimed to test the mid-term effects of body position on the resting state EEG activity, and the carry over effects of supine condition upon the return to sitting condition of the supine group, respectively. The source analyses carried out with standardized low resolution electromagnetic tomography (sLORETA) software allowed us to identify the cortical regions in correspondence of which groups differed in high-frequency EEG bands.

## Materials and Methods

### Participants

Thirty-two young undergraduates (mean age: 23 years, SD ± 1.5 years) of the University of Padova entered the experiment. All participants were native Italians, fully right-handed (on average 89.61 ± 7.13% according to the Edinburgh Handedness Inventory; Oldfield, 1971) females, and had normal or corrected-to-normal vision. None of the participants had been treated for any neurological or psychiatric disorder, nor were they under pharmacological treatment at the time of the experimental session. Every subject received a course credit for participating in the experiment. All students gave their written informed consent to the study, according to the Declaration of Helsinki. The experimental procedures were approved by the Ethics Committee of the School of Psychology, University of Padova.

### Task and Procedure

Participants were randomly assigned to the experimental (i.e., horizontal Bed Rest; *n* = 16) or control condition (i.e., Sitting Control; *n* = 16). The two groups had similar age, handedness, state-and trait-anxiety, forward and backward digit span, Raven IQ level and blood pressure (see details on **Table 1**).

**Table 1:** Behavioral characteristics of participants assigned to the experimental (hBR) and control (SC) group.

	hBR group	SC group	*t*(30)	*P*
Age	23.37 ± 1.96	22.44 ± 0.63	1.82	*ns*
Handedness	89.06 ± 6.11	90.16 ± 8.19	-0.43	*ns*
STAI-Y1	37.87 ± 6.45	37.12 ± 5.24	0.36	*ns*
STAI-Y2	37.87 ± 6.45	37.12 ± 5.24	0.36	*ns*
Forward digit span	6.31 ± 1.30	6.50 ± 0.73	-0.50	*ns*
Backward digit span	5.00 ± 1.09	5.00 ± 0.97	0.00	*ns*
Raven IQ (age-corrected)	118.31 ± 10.40	120.81 ± 10.13	-0.69	*ns*
Diastolic blood pressure	74.62 ± 6.61	75.44 ± 4.73	-0.40	*ns*
Systolic blood pressure	116.06 ± 8.42	117.69 ± 6.94	-0.59	*ns*

After participants were randomly assigned to the hBR or SC condition, they were prepared for electrophysiological recording. The experimental session started with the 3-min, open-eyes resting EEG recording acquired while participants of both groups sat on a soft chair (T0 interval; **Figure 1**).

Participants were requested to relax and refrain from moving. To limit eye movements during EEG recording in both SP and BR conditions, participants have been invited to watch a fixation cross in the center of a PC monitor. After this common baseline EEG measure, students assigned to the experimental hBR position laid on a mattress parallel to the floor, whereas those assigned to the control SC position maintained the position on the soft chair throughout the experiment. The second 3-min, open-eyes resting EEG was therefore recorded, i.e., in seated position for SC participants or in supine position for hBR group (T1 interval), with the same instructions. These two measures allowed us to compare the EEG activity of different groups assigned to specific conditions. In particular, the between-group contrast on T0 was aimed at excluding *a priori* EEG differences in the random assignment of participants to SC or hBR group, whereas the comparison of the next 3-min EEG (T1) was aimed at highlighting the effect of seated vs. supine/horizontal position on the resting state EEG activity. In addition, participants were asked to stay in that position, relaxed and with open-eyes, for 120 min, afterward another 3-min EEG was recorded (T2 interval; **Figure 1**). Finally, all participants regained the seated position, and the last 3-min rest open-eyes EEG was recorded (T3 interval). Measures T1 and T2 allowed us to investigate the mid-term 2-h effect of maintaining the body position on the EEG activity, whereas the last body transition T3 permitted to analyze the effects on EEG of returning in sitting position after being 2-h supine. The clear presence of blinking (which was subsequently well-corrected in the EEG analyses) during the four EEG acquisitions ensured that participants kept their eyes open; in addition, during the 120-min interval between T1 and T2, one of the experimenters remained in the participants’ room to make sure they did not fall asleep. To provide sufficient neck support during EEG acquisition by avoiding muscular artifacts due to the long experimental session, a U-shaped travel pillow was placed under the heads of participants. Seated participants were also allowed to rest their heads on the chair headrest during the 2-h period between T1 and T2^1^.

### Data Acquisition and Analysis

Electrophysiological activity was recorded by 38 tin electrodes, 31 mounted on an elastic cap (ElectroCap) according to the International 10–20 system (Oostenveld and Praamstra, 2001). The other seven electrodes were applied below each eye (Io1, Io2), on the two external canthii (F9, F10), nasion (Nz) and the mastoids (M1, M2). All cortical sites were on-line referred to Cz, and off-line re-referenced to the mean activity of the whole scalp by the average reference procedure. Data were stored using the acquire software NeuroScan 4.1 version (NeuroScan Labs, Sterling, VA, United States). Amplitude resolution was 0.168 μV/bin; the bandwidth ranged from DC to 100 Hz (6 dB/octave). The sampling rate was set at 500 Hz and impedance was kept below 5 KΩ. EEG was continuously recorded in DC mode and stored for later analysis. After data collection, the EEG signal was corrected for blinks and eye movement artifacts according to Ille et al. (2002) by BESA software (Brain Electrical Source Analysis, 5.1 version). Each EEG epoch was divided into 4096-ms time intervals, and thus included 44 samples with 0.244 Hz fast Fourier transform (FFT) resolution. Given the constraint of the FFT to use 2^n^ samples, the width of each interval was necessarily forced to 2048 samples, corresponding to a 4096-ms interval. Artifact rejection was performed on each epoch, with both amplitude and derivative (with respect to time) thresholds^2^ (250 mV and 100 mV/ms, respectively). The remaining epochs were then visually inspected for any residual artifact. On average, 15% of the trials were rejected evenly distributed among intervals. For each participant, and for each interval, the FFT was averaged across those epochs that, after windowing with a tapered cosine, were free of residual artifacts. The High-Beta band^3^ (20–35 Hz, effective β range: 20.25–35.13 Hz) and Gamma band (35–65 Hz, effective γ range: 35.38–49.04 and 51.24–65.15 Hz^4^) were analyzed. Based on the mean distribution of each band, electrodes were clustered into four regions of interest with two spatial factors consisting of two levels each: anterior–posterior asymmetry and laterality. Each quadrant therefore included the averaged amplitude of five electrodes: Anterior Left (AL: Fp1-F3-FC3-F7-FT7), Anterior Right (AR: Fp2-F4-FC4-F8-FT8), Posterior Left (PL: P3-P7-TP7-T7-O1), and Posterior Right (PR: P4-P8-TP8-T8-O2). This clustering allowed us to include, in our statistical analyses, most of the scalp activity, through the use, in agreement with our previous work (Spironelli et al., 2016), of 20 out of 30 electrodes placed on the left and right side of the cap. Individual ANOVAs were carried out for each EEG band including the factor Group (two levels: SC vs. hBR) and three within-subject factors: Interval (four levels: T0 vs. T1 vs. T2 vs. T3), Region (two levels: Anterior vs. Posterior), and Laterality (two levels: Left vs. Right hemisphere). *Post hoc* comparisons were performed using the Tukey HSD test (*P* < 0.05), and the Greenhouse–Geisser correction was applied when necessary, that is when variables with more than two levels were involved.

In order to identify neural sources underlying group differences in open-eyes resting EEG, the distributed source density solution of High-Beta and Gamma activity were separately carried out by sLORETA (Pascual-Marqui, 2002). Since sLORETA computes the smoothest possible 3D-distributed current source density solution constrained to gray matter, this approach was particularly suited for our analysis since it does not need an *a priori* number of known sources. Starting from all of the 4096-ms epochs available from each subject and interval – exactly the same which entered FFT analysis after artifact rejection – a single, 38 × 38 complex-valued, cross-spectral matrix for each participant, EEG band (i.e., High-Beta and Gamma) and experimental Interval (T0 vs. T1 vs. T2 vs. T3) was computed. All cross-spectral matrices were then converted in sLORETA transformation matrices to reduce the noise associated with measurement, to minimize the dependence of the source current density on individual subjects, and to eliminate components in the EEG spectra that were common to both groups. This transformation algorithm uses the three-shell spherical head model registered to the Talairach Human Brain Atlas (Talairach and Tournoux, 1988), available as MNI coordinates. The estimate of the source-current density in High-Beta and Gamma frequency range was carried out, for each interval in which previous ANOVAs revealed statistical between-group differences on FFT analyses, to determine which brain regions contained any significant differences. The method implemented by sLORETA software [i.e., the Statistical non-Parametric Mapping (SnPM)] yielded an adjusted statistic threshold to implement a multiple, pair-wise *t*-test on each element of the average source-current density vectors obtained from participants, in each band and interval. All results are expressed in Talairach coordinates (Talairach and Tournoux, 1988).

## Results

### FFT Spectral Analysis

#### High-Beta Band

**FIGURE 2:**
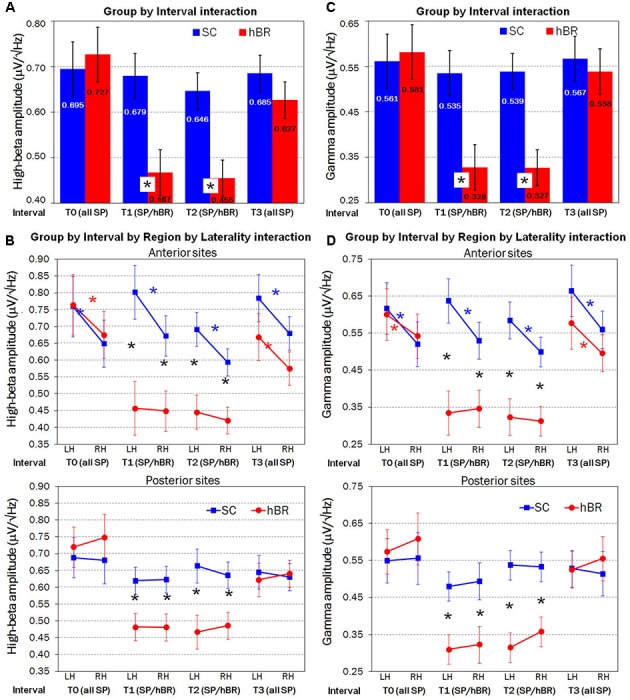
Fast Fourier transform (FFT) analyses carried out on SC (blue) and hBR (red) group. **(A)** Significant two-way Group by Interval interaction on mean High-Beta (β) amplitude. **(B)** Significant four-way Group by Interval by Region by Laterality interaction on mean β amplitude. **(C)** Significant two-way Group by Interval interaction on mean Gamma (γ) amplitude. **(D)** Significant four-way Group by Interval by Region by Laterality interaction on mean γ amplitude. SP, seated position; SP/hBR, seated position/horizontal Bed Rest; LH, left hemisphere; RH, right hemisphere. Asterisks indicate significant *post hoc* (Tukey HSD) differences.

Overall, β band amplitude was greater within the left rather than the right hemisphere [0.64 vs. 0.60 μV/Hz^2^; Laterality main effect: *F*(1,30) = 4.05, *P* < 0.05]. In addition, the within-subjects factor Interval was significant [*F*(3,90) = 15.92, GG ε = 0.73, *P* < 0.001], revealing that, regardless of group, β amplitude was lower in T1 relative to T0 interval (*P* < 0.001), whereas no difference was found between T1 and T2 intervals. Compared with T2, that is 2-h after hBR or SC assignment, in T3 participants showed a significant increase of β activity (*P* < 0.001). However, the two-way Group by Interval interaction [*F*(3,90) = 9.65, GG ε = 0.73, *P* < 0.001] revealed a different pattern of β band distribution in the SC and hBR groups. **Figure 2A** shows that the reduced β amplitude was observed in the hBR group only: indeed, SC participants showed no modulation across time in β distribution, whereas hBR group showed significantly lower β amplitude in T1 and T2 with respect to both T0 and T3 intervals (all *P*s < 0.001).

An important control condition is represented by the lack of between-group effects in β amplitude, during T0, that is, when all participants were in the seated position. Instead, as expected, in T1 and T2, hBR group revealed a significantly reduced β amplitude with respect to the SC group (both *P*s < 0.001). Again, no between-group difference was found in T3 interval, when groups were both in the seated position.

In addition, the four-way Group by Interval by Region by Laterality interaction [*F*(3,90) = 3.35, GG ε = 0.70, *P* < 0.05] revealed that between-group differences had a particular antero-posterior, lateralized distribution. **Figure 2B** shows that, in T0 interval, i.e., when all subjects held seated position, an anterior, left-lateralized (all *P*s < 0.001) pattern of activation marked participants’ EEG β activity, regardless of group assignment. This greater left vs. right anterior β amplitude was found in SC group across all four intervals (all *P*s < 0.001), whereas in hBR participants, during both T1 and T2 intervals, β amplitude was significantly reduced (all *P*s < 0.001) and the left asymmetry was abolished. When hBR regained again the seated position in T3, an anterior, left-lateralized pattern of activation was found (*P* < 0.001). Considering posterior sites, all groups revealed a clear bilateral distribution of β activity across all intervals, but again, during supine positions in T1 and T2, hBR women had significantly lower β amplitude with respect to the SC group (all *P*s < 0.001).

#### Gamma Band

The ANOVA carried out on Gamma EEG activity showed a pattern very similar to that of High-Beta band. The within-subjects Interval main effect [*F*(3,90) = 18.05, GG ε = 0.84, *P* < 0.001] revealed lower γ amplitude in T1 relative to T0 interval (*P* < 0.001), no difference between T1 and T2 intervals, and a significant increase of γ activity in T3 vs. T2 (*P* < 0.001). However, the two-way Group by Interval interaction [*F*(3,90) = 11.57, GG ε = 0.84, *P* < 0.001] showed that the reduced γ amplitude described in the Interval main effect was observed in the hBR group only (all *P*s < 0.001), whereas SC participants showed no modulation of γ amplitude across time (**Figure 2C**). In addition, the four-way Group by Interval by Region by Laterality interaction [*F*(3,90) = 3.05, GG ε = 0.79, *P* < 0.05] revealed the same anterior, left-lateralized distribution already found for β band analysis. **Figure 2D** shows, in T0 and T3, participants’ anterior, left-lateralized pattern of activation (all *P*s < 0.05), similar in both groups. This greater left vs. right anterior γ distribution persisted in SC group across all intervals (all *P*s < 0.001), whereas γ amplitude was significantly reduced (all *P*s < 0.001) and bilaterally distributed in hBR group during both supine intervals, T1 and T2. Concerning posterior sites, all groups revealed a stable bilateral distribution of γ activity, that was significantly lower in hBR compared to SC group again during T1 and T2 (all *P*s < 0.001).

### sLORETA Source Analysis

Fast Fourier transform analysis carried out on both High-Beta and Gamma bands revealed significant between-group differences throughout the 2 h of hBR supine condition. Therefore, source analyses comparing cortical distribution of the two groups were limited to these two intervals.

**FIGURE 3:**
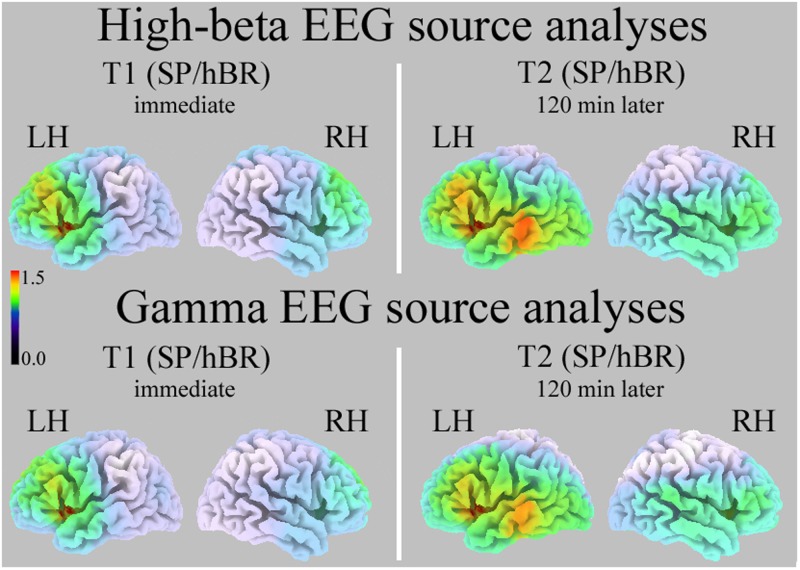
Standardized low resolution electromagnetic tomography (sLORETA) source analyses of High-Beta (Top) and Gamma (Bottom) EEG bands in the T1 (Left) and T2 interval (Right). Increasing intensities of red indicate significantly greater current density in SC than hBR participants (all *P*s < 0.01). SP/hBR, seated position/horizontal Bed Rest; LH, left hemisphere; RH, right hemisphere.

Standardized low resolution electromagnetic tomography computed on T1 and T2 intervals revealed a significant between-group difference in the High-Beta distribution (all *P*s < 0.01, two-tailed): SC participants had significantly greater High-Beta activity than hBR in the left insula/Inferior Frontal Gyrus (during T1, approximate coordinates: -38, 8, 4; during T2, approximate coordinates: -38, 12, 5; **Figure 3**, Top).

Standardized low resolution electromagnetic tomography carried out on EEG Gamma band during T1 and T2 intervals revealed a very similar significant between-group difference (all *P*s < 0.01, two-tailed): SC participants had significantly greater Gamma activity than hBR in the left insula/Inferior Frontal Gyrus (during T1, approximate coordinates: -38, 8, 4; during T2, approximate coordinates: -38, 12, 5; **Figure 3**, Bottom).

## Discussion

The present study aimed at analyzing the effects of body posture on high-frequency cortical activity (i.e., High-Beta and Gamma bands) in a sample of young healthy women using a mixed, between-subjects, repeated-measure experimental paradigm.

A first, main result revealed that the two groups exhibited similar levels of high-frequency EEG activity in the T0 interval, i.e., when all women where in a seated position, allowing us to exclude *a priori* EEG differences in participants’ random assignment to SC or hBR group. In the following 3-min EEG recording, the T1 interval, i.e., when hBR participants laid supine on a mattress parallel to the floor, SC showed an intact level of High-Beta and Gamma rhythms, compared with T0 interval, whereas hBR group exhibited a significant decrease of cortical activity, revealing the crucial effect of body position on the resting state EEG activity, in agreement with recent literature (Chang et al., 2011; Thibault et al., 2014). However, from past research it was not clear whether this effect is transient or long-lasting, and whether after regaining the sitting position the cortical activity was affected by carry over effects of the posture period. With respect to those studies that used brief body transitions (from a few to 15 min), we investigated also mid-term effects of supine position. Indeed, in many fMRI experiments, participants lay down for about 1 h, so we wished to measure cortical activity in this range of durations. The pattern of decreased activation was observed not only soon after the change from sitting to supine, but it lasted for the next 2-h (T2 interval). When the hBR group regained the sitting position during the T3 interval, it showed levels of high-frequency EEG activity similar to that observed in the T0 interval, and not different from that found in sitting controls. Therefore, the high-frequency bands showed a clear inhibition that started soon after the acquisition of the supine position.

The second, important result arose from the significant four-way interaction: both groups showed greater left vs. right anterior asymmetry when they were seated (i.e., in all intervals for SC, and T0 and T3 intervals for hBR participants), and bilateral posterior activity on both High-Beta and Gamma EEG bands. In addition, the source analyses carried out with sLORETA software confirmed that SC participants had significantly greater high-frequency activity (both High-Beta and Gamma EEG bands) than hBR group in the left insula/Inferior Frontal Gyrus, including left Broca’s area during T1 and T2 intervals. This result is important as clearly shows that supine posture involves the activation of cortical networks different from those involved in sitting condition. The involvement of different neural networks led us to hypothesize that different processes occur in individuals under the two postural resting state conditions. One limit of the studies using resting state is represented by the lack of behavioral and cognitive cues typically achievable with active tasks, which could help to interpret results. Therefore, the inner processes occurring in participant’s mind are hypothesized speculatively starting from the spatial distribution of the cortical regions activated during the resting state. According with Christoff et al. (2004), spontaneous thought processes occur frequently in resting state when no task is requested, and seems to be associated to bilateral Superior Temporal Gyri [Brodmann Areas (BA 38)] and left Middle Frontal Gyrus (BA 10/46) activation. Authors suggested that the temporal lobe activation reflects the long-term memory processes underlying the spontaneous flow of thoughts, that, according with Bookheimer’s (2002) and Hagoort’s (2005) model, should be unified in different portions of the left Inferior Frontal Gyrus. More in general, Koechlin and Jubault (2006) suggested that Broca’s area activation reflects inner speech or covert verbalization during task performance. In the present context, the activation of anterior sites located on frontal cortical regions may reflect participants’ inner speech/thinking during a resting condition. Another possible interpretation of subjects’ anterior left lateralization comes from Mazoyer et al. (2001) study, in which an extended fronto-parietal neural network was found while participants attained a conscious resting state. Authors suggested that this pattern may indicate activity of (episodic) working memory driven by emotion and supervised by the executive control of left prefrontal regions. Thus, in the present context, the activation of left frontal sites might also reveal participants’ executive linguistic control while they try to relax. Another interesting suggestion derives from the work of Szpunar et al. (2007) who tried to identify the neural mechanisms underlying the representations of the future. In that study, authors found that, among other areas, the left Middle Frontal Gyrus (BA 6/8) is critical for distinguishing future envisions from remembering past experiences. These results are in agreement with a very recent work of Lifshitz et al. (2017) in which MEG was used. For the Beta and Gamma 1 bands, similarly to our results, greater amplitude was found at parietal sites in sitting vs. supine participants, but there was more limited evidence of greater activation of left prefrontal areas in sitting participants. We interpret this result to be due essentially to the methods used. MEG is able to record mainly tangential electrical fields, and with greater accuracy from cortical surface than from deep sources; EEG is sensitive to both radial and (to a lesser extent) tangential electrical fields and it’s a technique able to detect also deeper sources. The two methods are not equivalent in capturing the electrical sources; rather, they provide complementary spatial information (Aydin et al., 2015). Indeed, the opercular frontal area and the underlying deep cortical insula represent large surfaces parallel to cortex, and are able to provide a relevant EEG signal, but the same sources, due to the orientation of the electrical fields, probably are not easily detected by the MEG sensors.

The present study, in line with past consistent literature, confirmed for high-frequency EEG bands and mid-term (2-h) postural duration, the cortical de-activation effects induced by the supine position. The reduced cortical activation involved both frontal and parietal sites and was promptly reversed when bedridden participants regained the sitting position. But our results further add to the current literature by evidencing how, during horizontal posture, participants lost the left frontal asymmetry exhibited during the sitting condition, a result pointing to the greater involvement, in the sitting condition, of linguistic-executive, left-lateralized neural networks probably related to inner speech/thinking. In humans, who are active and awaken when standing or sitting, the supine position prepares the body and the brain for sleep, with increasing amplitude of slow EEG rhythms, decreased amplitude of high-frequency cortical rhythms, and reduced left frontal asymmetry. The physiological mechanisms leading to the observed neural functional changes are still unknown: carotid baroreceptors, sensitive to change of posture at cardiovascular level, are also able to influence cortical activation, therefore they are among the possible candidates playing a role in the observed mechanism (Dworkin et al., 1994; Angrilli et al., 1997).

## Conclusion

The present experiment confirmed past research showing, in the supine position, a clear decreased amplitude of the high-frequency EEG bands typically associated with cognitive arousal. In addition, compared with sitting participants, the observation of a substantially weakening of the networks activated in the left prefrontal sites and insula, presumably related to a decreased inner speech and thoughts, provides some hints on the important changes induced by body position in the research carried out with fMRI and PET, in which participants lie down horizontally. In past research on body position (Spironelli and Angrilli, 2011, 2017; Fardo et al., 2013; Messerotti Benvenuti et al., 2013), the Bed Rest position induced a significant change also in the networks involved in emotion, pain perceptual and word learning-recognition tasks. Therefore, the present results, together with past research, led to the important conclusion that, both in resting state and in passive perceptual tasks, the horizontal position – typical of fMRI and PET research – leads to a substantial change of the neural networks usually activated in awaken sitting subjects, a result which cannot be ignored when specific hypotheses are put forward and compared across studies with similar paradigms and stimuli, but different body positions and neuroimaging methods. Indeed, our results have an impact especially on important theories postulating the involvement of specific neural networks, typically built up starting from supine studies, and for which the variety of responses achieved depending on posture necessarily relativizes the relevance and the ecological validity of the study itself. This hopefully will stimulate a scientific debate on an important variable, the body posture, neglected so far in neuroscience research.

## Author Contributions

AA and CS contributed to the conception and the design of the work. CS acquired and analyzed the data, wrote the draft of the work. AA revised the draft. CS and AA approved the final version of the work and agree to be accountable for all aspects of the work in ensuring that questions related to the accuracy or integrity of any part of the work are appropriately investigated and resolved.

## Conflict of Interest Statement

The authors declare that the research was conducted in the absence of any commercial or financial relationships that could be construed as a potential conflict of interest.

## Footnotes

1 To exclude that EEG results might be affected by significant muscular activity, we have carried out a preliminary analysis of the 80–100 Hz EEG band. Since EMG is characterized by a very broad spectral range going from 20 to over 300 Hz (Cacioppo et al., 2007), if muscular artifacts played an important role in the EEG results, then the same results and patterns should be found also in the higher bands (e.g., 80–100 Hz) best representing the muscular activity. ANOVA carried out in the 80–100 Hz range did not reveal neither a main effect nor an interaction including the Group factor, thus confirming the muscular artifacts not to be involved in the significance of the present EEG results.

2 Using BESA software, artifact rejection includes an Amplitude threshold criterion, that rejects the trial within which the difference between the maximum and the minimum amplitude is exceeded, and a Derivative threshold criterion, that rejects each trial within which the largest amplitude difference between two adjacent time samples is exceeded.

3 For each EEG band, we indicated the typical frequency range and the “effective range,” that is the real width of each EEG band.

4 Gamma amplitude was obtained by the sum of all values within the gamma spectral band excluding the lines around 50 Hz frequency to avoid possible noise artifact due to mains power.
